# Sludge, dark patterns and dark nudges: A taxonomy of online gambling platforms' deceptive design features

**DOI:** 10.1111/add.70085

**Published:** 2025-04-29

**Authors:** Philip Newall

**Affiliations:** ^1^ School of Psychological Science University of Bristol Bristol UK

**Keywords:** behavior change, behavioral science, deceptive choice architecture, gambling platforms, nudge, online gambling

## Abstract

**Background:**

Gambling research has highlighted various aspects of deceptive design. For example, land‐based casinos are physically designed to encourage people to gamble there for longer. Similarly, various electronic gambling products, such as electronic gaming machines, exhibit “structural characteristics” that likely encourage people to continue using them. Here I argue that the deceptive design of online gambling platforms is an important yet relatively neglected topic, representing the digital equivalent of land‐based gambling venue design. This topic's importance is underscored by online gambling's international growth.

**Argument:**

Online gambling platforms' deceptive design features can be categorized using three terms from the interdisciplinary field of behavioral science. “Sludge” refers to detrimental frictions, which feature for example in online gambling platforms' withdrawal processes. “Dark patterns” refers to deceptive user‐interface design more broadly, which includes sludge‐based features and other design features such as high suggested deposit/bet sizes. Finally, “dark nudges” refers to all of these as well as other aspects of deceptive design, such as presenting gambling as a fun activity.

**Conclusions:**

The complexity of online gambling platforms poses challenges for researchers, to understand what effects various design features have on behavior, and also for policymakers, to ensure fairer outcomes for people. Increased awareness and collaboration are needed from many stakeholders to better understand deceptive design features' behavioral impacts and to give them the appropriate regulatory attention in comparison to other determinants of harm.

## INTRODUCTION

Deceptive design features have been highlighted across different areas of gambling research. Land‐based casino design, for example, is based on creating smooth and disorienting maze‐like spaces, containing secluded areas directing people toward electronic gaming machines (‘EGMs’; [1]), which are one of the casino's most profitable yet harmful products [1, 2, 3, 4]. Although EGMs are programmed to pay‐out less than is wagered on them, various ‘structural characteristics’ arguably encourage people to continue using them. Previously established EGM characteristics include their fast speed and immersive audiovisual features [5, 6, 7], their use of nearly winning ‘near miss’ outcomes [8, 9, 10] and the tendency for ‘multi‐line’ slots to celebrate losing outcomes [11]. Similar product‐based structural characteristics can be observed in on‐line gambling products, both in online slots [12] and also in online sports betting [13]. Here, I argue that the deceptive design of online gambling platforms is an important yet relatively neglected topic, representing the digital equivalent of land‐based casino design.

A recent meta‐analysis of international prevalence studies suggested that gambling online was, out of the 57 risk factors tested, the strongest predictor of gambling‐related harm [14]. Compared to land‐based gambling, online gambling can be accessed at any time of day [15] and from numerous locations via mobile devices [16]. Furthermore, a greater range of gambling formats is often available in on‐line gambling compared to what can be found on EGMs [17], and engagement with multiple formats is itself a risk‐factor for harm [18]. It is perhaps even easier for someone to switch from a slots product to a poker table in online gambling, compared to in a land‐based casino. Many international jurisdictions, including Brazil and various Canadian and United States states, are increasing the legal availability of gambling, specifically via online and app‐based products [19, 20, 21]. Cryptocurrencies can also be used as payment solutions for online gambling, which can facilitate access even in jurisdictions without legal markets [22]. Online gambling is seen as a key driver in the international gambling industry's growth, which is forecast to reach $700 billion in annual profits by 2028 [23].

An understanding of online gambling platforms' deceptive design does not need to begin in a vacuum, but can use concepts from the interdisciplinary field of behavioral science. Much of behavioral science research focuses on beneficial changes to design environments called ‘nudges’ [24, 25, 26]. Auto‐enrolling workers into pensions is seen as one classic beneficial nudge, given the pervasive impact of default options on choice [27] and people's tendency to under save for retirement [28]. However, in recent years, attention has turned toward how online environments in particular can be designed in ways that make people worse‐off [29, 30, 31, 32, 33, 34], called deceptive design here, for which three main terms have been used in the behavioral science literature.

The first established term, ‘sludge’, was coined by the originators of nudge [32, 35]. As helpful nudges often act to make some beneficial action easier to perform, sludges act in reverse to this, by instead making that beneficial action harder to perform [35]. Examples of sludge include excessively complex forms to claim some government benefit or the use of deliberately long phoneline waiting times required to cancel a recurring subscription. ‘Dark patterns’ is the next established term, which originates from the user experience (UX) design community [36]. This term refers to a range of tactics that website and app designers can use to trick people into taking actions that are more profitable for the website [37], such as by automatically signing them up for unwanted recurring subscriptions. Importantly, within the context of online gambling, this term includes tactics that could also be considered sludge, such as tactics used to make sensible deposit limits harder to set [38]. The third and final established term, ‘dark nudges,’ is a term that is intended to be as wide‐ranging as ‘nudge’ [39]. Nudges can in principle use any tactic [24, 25, 26], with their core attribute being that they should help make people better‐off. Dark nudges can similarly in principle use any tactic, with their core attribute being the tendency to make people worse‐off [39]. Dark nudges can, therefore, be seen as including the tactics highlighted by the first two terms, as well as others, such as the alcohol industry's presentation of drinking as a normal leisure activity [40, 41].

As these terms come from a large interdisciplinary field, they are not mutually exclusive, and different terms can often be accurately used to describe the same aspect of deceptive design. Given this fact, I have attempted to present the terms logically, by beginning with the most restrictive term and then focusing on the additional aspects of deceptive design that can be understood within the more inclusive terms. As all three terms are in active use across a large field, it would be inappropriate to use only one.

Online gambling may be a particularly relevant domain for deceptive design for three reasons. First, behavioral science has as a field revealed numerous judgmental biases [42], such as the ‘anchoring’ effect where judgments of magnitude are affected by incidental numbers in the environment. As gambling involves the risking of money, these judgmental biases could well be taken advantage of in online gambling platforms. Second, the design of online environments can be honed to exploit these biases over time, as small‐scale ‘A/B test’ trials can be run in‐house to detect effects on gambler engagement and losses. Third, while consumer welfare might be hard to measure in many domains, thereby leading to potential disagreement over whether various design choices are beneficial or deceptive, gambling harms are well‐established [43, 44, 45], with the risk of harm increasing with gambling losses [46, 47].

The present work is not intended as an exhaustive list based on database searches and is instead a personal perspective from a researcher with a history of working in this area, for the following reasons. Many competing terms have been used in this area, and relevant research could well use any or even none of these terms when interpreting empirical findings. Much relevant research has been published as gray literature, for example, as peer‐reviewed reports for funders, or published just as website blogs, both of which might not appear in database searches. As with other aspects of gambling research, this could be seen as a fast‐moving area, meaning that any exhaustive review may well soon become out of date regardless. Instead, the present taxonomy is intended more as a categorization tool, to help readers to understand how these terms from the behavioral science literature relate to one another and is based on the author's knowledge developed from contributing to this area. Hopefully, the merits of this approach can be supported by the number of below‐reviewed findings, which did not appear in a recent keyword‐based scoping review of this area [48].

## SLUDGE

As a term, sludge is generally used to refer to detrimental frictions that make it harder to perform some beneficial action [32, 35, 49], which is the definition used here.

Online gambling websites usually allow people who gamble to deposit and hold money in an account, with this account balance representing the amount of money that can be gambled at any time. Because this account balance could, therefore, represent a tempting invitation to gamble, it is generally considered that promoting the ease of withdrawals would be a beneficial nudge (i.e. the opposite of a dark nudge; [50, 51, 52]). However, online gambling websites can implement sludge‐based tactics to instead inhibit withdrawals relative to deposits [51]. First, deposit pages might be accessible via fewer clicks from the homescreen than corresponding withdrawal pages. This measure, the ratio of clicks to take one action compared to its mirror image (often account creation vs. deletion), is one measure of sludge that can be used across multiple industries [37]. For example, one gambling‐like investment app required nine clicks for account deletion versus five clicks for account creation [37]. Furthermore, it has also been qualitatively observed that this same pattern holds for regulated gambling websites as well [53], with one study suggesting that sign‐up can be done in under 5 minutes, with account closure often involving lengthy calls with customer service [54]. However, as will be highlighted in the Discussion below, the extent to which these tactics influence behavior is unclear.

Great British (GB) gambling websites have also in the past taken sludgy friction further, by processing deposits instantly, but by placing delays of up to several days on withdrawals. Furthermore, during this withdrawal‐delay prominent buttons were displayed to encourage gamblers to reverse the withdrawal, and thereby instantly enable further gambling with the money that was intended to be withdrawn. Although these ‘reverse‐withdrawals’ are now banned by the GB regulator, they can still be performed on gambling websites elsewhere [55]. Numerous sludge‐based tactics can, therefore, absent regulation, be used to make withdrawals and account closure unnecessarily hard in online gambling.

Online gambling websites are generally required to provide safer gambling tools to help gamblers monitor and control their amount of time and money spent gambling [56, 57]. Deposit limits, for example, allow gamblers to set binding upper bounds on how much they can deposit in a given period of time. The setting of sensible deposit limits is, therefore, a clear beneficial action for online gamblers. However, deposit limits have been shown to display unrealistically high deposit limits as suggested amounts (sometimes as high as six‐figures), with gamblers either having to scroll down dropdown menus containing many unrealistically high amounts, or proceed via a further click to enter their own preferred limit [38]. Results of a randomized‐controlled‐trial (RCT) showed that the unrealistically high suggested amounts led to higher deposit limits than a simple text‐entry box without any suggested amounts [38].

Last, the regulator of the GB online gambling market says that information about gamblers' chances of winning ‘must be easily available’ on chance‐based online gambling games [58]. The clear provision of this information should help people to make informed choices. However, in an audit study of over 300 roulette games, the mandated information was found randomly placed in dense information screens with an average of over 2000 words each and were almost always shown in the smallest font size on the screen. However, there is at present no evidence on what effect on behavior better information provision might have, existing evidence on, for example, calorie labelling suggests that any effects might be limited in size [59].

## DARK PATTERNS

Dark patterns refer to deceptive user‐interface design features of websites [37]. Because online gambling is an inherently web‐ and app‐based environment, the examples of sludge discussed above could also be considered as dark patterns as well [36]. However, unlike sludge, dark patterns do not need to focus solely on making beneficial actions harder to perform, they can instead, for example, use deceptive design features to increase the salience of options that might be more profitable for the gambling platform.

One tactic that could also be described as either sludge or a dark pattern is the tendency to make safer gambling information less prominent. However, because many of these tactics use a range of user‐interface design tricks, they are perhaps more similar to the other examples in this section. One study of GB‐regulated gambling websites found that 30% placed the links to safer gambling in a font color with minimal contrast against the overall background, making the text hard to spot [60]. The safer gambling pages themselves could also be text‐heavy and tended to avoid the salient and colorful buttons found on the websites' gambling pages. Furthermore, this same study found that 90% of the gambling websites' search bars would not redirect to relevant safer gambling tools when the word ‘limit’ was searched. However, this tendency can get even more extreme when considering gambling websites that accept deposits in cryptocurrencies—a way of depositing money in online gambling that is not allowed by the GB regulator [22]. Many (22.5%) of these cryptocurrency‐based gambling websites had safer gambling pages containing conventional promotions encouraging gambling, such as deposit bonuses. Furthermore, 7.5% of cryptocurrency‐based websites' safer gambling pages were completely unlinked to on the gambling website itself, with pages being found only via search‐engine or with links being provided only after a conversation with customer support. Although relatively under‐researched, this suggests that even more extreme instances of deceptive design might be found on cryptocurrency‐based gambling websites.

Another tactic is a tendency for gambling websites to suggest specific deposit amounts, which may well be higher than gamblers' average deposits [51]. This tactic does not fit under the most commonly used definition of sludge, because it does not focus on friction and is more about directing people toward the most profitable option for the gambling website. One study found that 30% of audited gambling websites had suggested deposits above the minimum size [53]. This same study found other dark pattern tendencies too. Overall, 70% of gambling websites had suggested in‐game bet sizes that were greater than the minimum allowed. Furthermore, reality checks, which are safer gambling tools that prompt gamblers to take a break after a given amount of time spent gambling, were in 60% of websites set by default to the longest and, therefore, least‐effective delay of 4 hours.

Finally, some research has explored aspects of dark patterns within contract for difference (CFD) trading apps [61]. CFDs can be considered a gambling‐like investment product because the disclosures that financial regulators require for them show that up to 89% of CFD trading accounts lose money [62]. However, these disclosures are shown with suboptimal levels of prominence, with only two of 14 audited mobile apps providing disclosures within the apps' main tabs, while two further apps had no disclosures that could be found in the user journey at all [61]. CFD websites tend to have easy to open demo accounts, which allow users to explore trading with play money. On opening a demo account, most apps sent frequent emails and mobile push notifications, which acted as salient reminders for the user to sign up for an actual trading account. However, it is unknown how much of an effect any of these dark patterns might have on behavior, which is a topic that will be returned to in the Discussion.

## DARK NUDGES

Dark nudges refer to any type of design feature that makes people worse‐off [39]. The breadth of this term means that it can, in online gambling, include the previously reviewed examples of sludge and dark patterns, as well as other tactics. The rest of this section will, therefore, focus on aspects of online gambling platform deceptive design that fit exclusively within this wider purview of dark nudges. Oftentimes, the tactics that are exclusive to dark nudges take advantage of some judgmental bias originating from the behavioral science literature [42].

The ‘affect heuristic’ is a widespread judgmental bias, where people substitute current feelings instead of rational reflection of risk and potential reward [63]. Gambling websites' tendency to present gambling as a fun and harmless activity can, therefore, be seen as a dark nudge [64]. In 2014 and in response to increasing public concern around the volume of gambling advertising, a group of large GB‐regulated operators started showing their own safer gambling message, ‘when the FUN stops, stop’ on their adverts, in their betting shops and on their websites [65], and this message has also been used in Las Vegas casinos [66]. Although this message was claimed to be a warning about gambling's potential harms, it also presents gambling as fun, and presumably, harmless for many. Independent testing of this message shows that it does not reduce contemporaneous levels of gambling and may actually lead to people gambling slightly more than if no message is shown at all [66]. Similar wording has been found in websites' safer gambling pages (e.g. ‘we want the gaming experience to stay fun’ [60]). Even less appropriate ways of presenting safer gambling information can be found on unregulated cryptocurrency‐based gambling websites, ‘Playing with [this operator] can be an enjoyable form of entertainment and You might even win some money. But You cannot win every time [sic].’ [22]. These are numerous examples of how gambling can be presented as being largely harmless, despite substantial evidence to the contrary [43].

Furthermore, a judgmental bias called ‘framing’, where the presentation of numerical information matters [67], is also relevant to the study on mandated information in GB‐regulated websites that was described previously in the sludge section [58]. Mandated information was always shown in what is called the ‘return‐to‐player’ format, which frames gambles in terms of gamblers' average winnings (e.g. ‘this game has an average percentage payout of 90%’). However, research has shown that the equivalent ‘house edge’ format, which frames gambles in terms of what gamblers' can expect to lose on average (e.g. ‘this game keeps 10% of all money bet on average’), is understood better and results in lower perceived chances of winning [68]. Therefore, a return‐to‐player of 90% and a house edge of 10% are two ways of framing the same information. Although the house edge is among the formats allowed by the regulator, none of the audited games used this better understood information format [58].

Another judgmental bias is the ‘conjunction fallacy’, where people overestimate the probability of a combination of events, if one or more of these events is ‘representative’ or easy to imagine happening. In the classic demonstration of this, participants read a description of ‘Linda’, which indicated her concerns for social justice, and subsequently rated that it was more likely that Linda was a bank teller who was active in the feminist movement, than it was for her to be a bank teller (active in the feminist movement or otherwise; [69]). In this case, the conjunction was judged as being more likely because it is representative of an easily imaged person. Similarly, advertisements for GB‐regulated gambling websites have been shown to involve complex soccer bet conjunctions (e.g. ‘Brazil to win, Neymar to score, both teams to score, and Xhaka to be carded’ [70]). These individual events are selected in gambling adverts to be representative, with for example Brazil being the team that have won more men's World Cups than any other and Neymar being the most expensive men's player in history. These representative conjunctions provide high potential wins, but rarely happen exactly, providing gambling websites with much higher profit margins than ordinary soccer bets [71]. Finally, gambling websites have tools allowing gamblers to create their own personalized versions of these complex bets [72]. This personalization potentially harnesses the illusion of control, another judgmental bias with substantial association with gambling research [73], which suggests that people are biased toward gambles involving some aspect of personal control.

One last relevant judgmental bias is people's inability to mimic random sequences, for example, for coin flips there being a tendency to underestimate the number of times that one side of the coin will be flipped in a row [74]. In gambling, this inability leads most prominently to the ‘gambler's fallacy’, where people tend to gamble against the continuation of a streak of identical outcomes [75]. Many electronic gambling products now display information about past random outcomes, such as in roulette [76]. Although research on the extent to which this information is provided in online gambling may be lacking, past outcome information does not help gamblers at all in random gambling formats such as roulette, so this practice is best explained as another exploitation of a judgmental bias. Research on the extent to which these and other potential dark nudges affect behavior is lacking, and therefore, this should be considered a priority for those working on this topic.

## DISCUSSION

The present taxonomy aimed to show the relevance of multiple aspects of deceptive design to online gambling and the overlap between them. As Table 1 shows, deposit limit‐setting tools [38], for example, make appropriate low deposit limits harder to select (sludge) via various aspects of their user‐interface design (dark patterns), while using the judgmental bias of anchoring (a dark nudge). Two other examples of deceptive design are also included in Table 1, to demonstrate the other categories reviewed in this taxonomy. While I have argued that the deceptive design of online gambling platforms is an important and understudied analogue of previous work on the physical design of land‐based casinos [1], this is a topic that also raises unique issues for researchers and policymakers.

**TABLE 1:** Three deceptive design features from online gambling platforms and how they relate to the three terms from behavioral science.

Design feature	Sludge—detrimental frictions	Dark patterns—deceptive user‐experience design	Dark nudges—any aspect of deceptive design, including the exploitation of judgmental biases
Deposit limit setting tools [38]	Additional clicks and/or scrolling required to set lower beneficial deposit limits	Suggesting higher deposit limits is more profitable for the website	Uses the ‘anchoring’ bias, that judgments are affected by numbers in the environment [42]
High suggested deposit and bet sizes [53]	Not applicable	Suggesting that users select an option that is more profitable for the website is a common dark pattern	Uses the ‘default’ effect, that suggested options are commonly followed [27]
Presenting gambling as a fun activity [60]	Not applicable	Not applicable	Uses the ‘affect’ heuristic, where current feelings are used to judge risk [63]

One issue regards what actions policymakers might choose to take in relation to these topics. The 2023 United Kingdom (UK) Government White Paper on gambling cited in particular the Behavioural Insights Team's 2022 audit [53, 77]. Relying on industry self‐regulation may not prove effective, because this has, for example, not meaningfully reduced the frequency of UK gambling marketing [78, 79, 80]. Another approach could be to mandate specific aspects of online gambling platform design, such as requiring that bet and deposit sizes are defaulted toward the minimum possible. However, regulation is slow in comparison to the gambling industry, which can constantly innovate new practices. It is hard to say whether this ‘whack a mole’ approach could yield meaningfully safer online gambling environments. Another more radical approach could be to implement an independent and universal system for processing online gambling payments, which could then collaborate with the research community to test and improve various safer gambling features via RCTs [81]. However, this system might be too costly or technically challenging to implement.

### Moving the evidence base forward

While the research reviewed here highlights a number of ways in which online gambling platforms appear to be designed in deceptive ways, there is a major limitation regarding our understanding of the extent to which they influence behavior. Some design features that appear unfair might have rather trivial impacts on behavior, while other features that the research community are unaware of may plausibly have more significant effects.

Most of the studies reviewed here are unable to estimate behavioral impacts. Audit studies can only record aspects of online gambling platforms that researchers are aware of and are able to systematically code. Furthermore, audits can be time‐intensive to execute, can usually only audit a subset of relevant websites or features and require repetition to ensure continuing relevance. Researchers could create complex online experiments that would be able to manipulate relevant design features to begin exploring potential causal effects. However, it would be costly and ethically challenging to attempt this task at scale. Furthermore, experimental gambling research has long been known to struggle to reproduce the affective responses of naturalistic gambling [82], meaning that this process might still be unable to produce concrete knowledge about online gambling behavior.

RCTs are generally seen as a gold standard methodology in gambling research, yet only one of the reviewed studies used this methodology [38]. While that study found significant effects in terms of the deposit limits set, it was likely underpowered to meaningfully detect potential subsequent changes in gambling behavior. A wider spread of stakeholder engagement is, therefore, needed to improve the quality of this evidence base. Journalists are one group who could help to raise further awareness and have previously brought attention to deceptive design features of investment trading apps [83, 84]. Online gambling platforms could, for example, increase the credibility of their commitment toward safer gambling by agreeing to execute RCTs on aspects of their design that independent experts deem to be potentially deceptive.

## CONCLUSION

In conclusion, the present work provided a taxonomy of deceptive design in online gambling, which aimed to increase awareness and promote informed debate around these features' potential detrimental impacts on gambling harms, as well as help stimulate future work exploring their potential behavioral effects.

## DECLARATION OF INTERESTS

P.N. is a member of the Advisory Board for Safer Gambling—an advisory group of the Gambling Commission in Great Britain. In the last 3 years, P.N. has contributed to research projects funded by the Academic Forum for the Study of Gambling, Alberta Gambling Research Institute, BA/Leverhulme, Canadian Institute for Health Research, Clean Up Gambling, Gambling Research Australia, NSW Responsible Gambling Fund and the Victorian Responsible Gambling Foundation. P.N. has received honoraria for reviewing from the Academic Forum for the Study of Gambling and the Belgium Ministry of Justice, travel and accommodation funding from the Alberta Gambling Research Institute and the Economic and Social Research Institute, and open access fee funding from the Academic Forum for the Study of Gambling and Greo Evidence Insights.

## Data Availability

Data sharing not applicable to this article as no datasets were generated or analysed during the current study.
